# Ovarian Stimulation Affects the Population of Mouse Uterine NK Cells at Early Pregnancy

**DOI:** 10.1155/2013/182531

**Published:** 2013-11-17

**Authors:** Parvin Dorfeshan, Mojdeh Salehnia, Seyed Mohammad Moazzeni

**Affiliations:** ^1^Anatomy Department, Tarbiat Modares University, P.O. Box 14115-111, Tehran, Iran; ^2^Immunology Department, Tarbiat Modares University, Tehran, Iran

## Abstract

The aim of this study was to determine the influence of ovarian stimulation on endometrial mouse NK cell population. For superovulation, the female adult NMRI mice were injected i.p. with 10 IU of the pregnant mare serum gonadotropin followed 48 h later by an i.p. injection of 10 IU human chorionic gonadotropin hormone. Ovarian stimulated and nonstimulated mice were mated with fertile male. The presence of vaginal plug proved natural pregnancy, and this day was considered as day one of pregnancy. Tissue samples were prepared from the uterine horn and spleen of both groups of study on 7th day of pregnancy. Serum estradiol-17*β* and progesterone were measured at the same time. The tissue cryosections were prepared and double stained for CD 161 and CD3 markers, and NK cells population was analyzed. Relative frequency of NK cells was significantly lower in stroma and myometrium in hyperstimulated mice compared with the control group. However, no difference was seen in percentage of NK cells in spleen. The ovarian stimulation influences the proportion of uterine NK cells and may affect the embryo implantation.

## 1. Introduction

Embryo implantation and maintenance of pregnancy are complex processes. The dialogue between maternal and fetal tissue possesses a critical role in establishment of pregnancy. Indeed the interactions between the trophoblast cells and the maternal immune cells including T cells, natural killer cells (NK cells), macrophages, and dendritic cells within the decidua are essential for placental development and successful pregnancy [1, 2]. Some changes in phenotypes and functions of the immune cells in implantation site at maternal and fetal interface were reported in pathological conditions such as recurrent spontaneous abortions and preeclampsia [3–7].

NK cells are one of the most important cell populations of innate immune system with diverse effectors functions like killing the tumor cells and virus-infected cells as well as secreting various cytokines. Their functions are finely regulated by the balance between a variety of activating receptor signals and inhibitory receptor signals which are triggered by specific major histocompatibility complex (MHC) or non-MHC ligands. NK cells are the main population of lymphocytes in the endometrium and especially early pregnancy decidua. Decidual NK cells produce a number of cytokines and other secretory factors that help trophoblast proliferation and differentiation and promote pregnancy success [8, 9]. 

The number of the uterine NK cells was changed within the reproductive cycle and pregnancy [10, 11]. In mice, uterine NK cells which are NK1.1^+^ appear in decidua following implantation [12, 13].

It was shown that both mouse and human uterine NK cells have angiogenic functions by producing and secretion of vascular endothelial growth factor and other angiogenic molecules [14–17]. Considering the cyclic changes in the population of NK cells of uterus it seems that their distribution and functions are under the control of ovarian hormones [18, 19].

In fact, estrogen and progesterone receptors have been detected on uterine NK cells [20–24], although there is some controversy in their presence according to the type of receptors, tissue source of the NK cells, and techniques of assessments [20–26].

Ovarian stimulation has been used to obtain the maximum possible number of oocytes in assisted reproductive techniques [27]. It was shown in many investigations that ovarian stimulation affects both the embryo quality and the uterine microenvironment [28–31]. Moreover the latter could affect the embryo development and implantation. Following the ovulation induction the ovarian hormones are produced at the supraphysiological level and the impact of high level of ovarian steroid hormones on different parameters of endometrium was shown by others [32–34]. However some investigations have focused on the effects of ovarian stimulation on population changes of the immune cells within the peripheral blood or uterine tissue, but there is some controversy regarding the obtained results [27, 35–38]. 

Lukassen et al. [37] used flow cytometric analysis to study the NK cell subsets of endometrium and showed that hormonal stimulation for in vitro fertilization (IVF) treatment positively affects the CD56^bright^/CD56^dim⁡^ NK cells ratio of the human endometrium by a relative decrease in the cytotoxic CD56^dim⁡^CD16^+^ NK cells number during the implantation window. 

Junovich et al. [38], however, demonstrated the decrease in frequency of total and regulatory endometrial NK cells during implantation window in ovarian stimulated samples.

Collectively, to our best knowledge, poor information is available regarding the effect of ovarian stimulation protocol on the proportion of NK cells within the uterus tissue; thus this study was undertaken to find the effect of hormonal stimulation of the ovary during IVF treatment on the distribution and number of the endometrium NK cells population in early mouse pregnancy. Splenic NK cells were also studied as a representative of their systemic changes following ovarian hyperstimulation.

## 2. Material and Methods

### 2.1. Animals

Adult female (8–10 weeks old) and male (8–12 weeks old) NMRI mice were cared and used according to the guide for the Care and Use of Laboratory Animals of Tarbiat Modares University and housed under a 12 h light : 12 h dark cycle.

### 2.2. Experimental Design

The adult female NMRI (National Medical Research Institute) mice were divided into two groups of study. 

*Control.* in this group the female mice (nonstimulated) were mated with a fertile male. The presence of vaginal plug proved the natural pregnancy and this day was considered as day one of the pregnancy. 

*Hyperstimulated Group.* the adult mice were superovulated with an i.p. injection of 10 IU pregnant mare serum gonadotropin (PMSG, Folligon, Intervent) followed 48 h later by an i.p. injection of 10 IU human chorionic gonadotropin hormone (HCG, Sereno) for ovulation induction. These mice were mated in the same manner of control group.

### 2.3. Blood Sampling and Hormonal Assay

To monitor the effect of PMSG and HCG treatment on plasma estradiol-17*β* and progesterone concentrations, the blood samples were obtained from pregnant mice in both groups on day 7 of pregnancy. The sera were then collected and stored at −20°C until the hormones assay. The concentration of progesterone and estradiol-17*β* in sera was measured with enzyme-linked immunosorbent assay (ELISA) method (Diaplus, USA).

### 2.4. Tissue Preparation

Five pregnant mice were sacrificed by cervical dislocation in each group of study on 7th day of pregnancy. Their uterine horns were exposed from any fat and mesentery; then the tissue samples were randomly prepared from different parts of uterine horns (at least from 6 different parts of uterine horns of each mouse) and spleen as a systemic lymphoid organ. Frozen sections of 5 *μ*m thickness were prepared (at least 5 sections from each tissue block) and fixed in cold acetone (Merck, Germany). The tissue sections were kept at −80°C until morphological and immunohistochemical evaluations.

### 2.5. Morphological Staining

To evaluate the morphology of the tissues, the sections were stained by routine hematoxylin and eosin technique and examined under light microscope.

### 2.6. Immunohistochemical Staining

The cryosections reached the room temperature and the staining area was limited by Dako pen (Dako, Denmark). The tissue sections were treated sequentially with protein block serum-free solution (Dako) for 10 min, serum blocking buffer containing 2% rabbit and goat serum for 30 min, and one drop of streptavidin (Dako) for 15 min followed by one drop of biotin (Dako) for 15 min (to neutralize the endogenous biotin). Then, the samples were incubated overnight with goat anti-mouse CD161 antibody (Santa Cruz Biotechnology Inc., USA) at 4°C followed by biotin-conjugated rabbit anti-goat antibody (Invitrogen, Germany). The samples were later treated with streptavidin-conjugated alkaline phosphatase (Roche, Germany). To block free sites of biotin or streptavidin and prevent their interfering with the next staining step, one drop of streptavidin and one drop biotin were added to samples separately and the samples were incubated for 15 min in each step. Then, the specimens were treated with alkaline phosphatase blue substrate (Vector Laboratories, USA) and levamisol as the inhibitor of endogenous enzyme. As the stains appeared, the reaction was stopped by washing the slide under tap water and rat anti-mouse anti-CD3 antibody (Pharmingen, USA), 0.3% H_2_O_2_ solution (to neutralize endogenous peroxidase), biotin-conjugated goat anti-rat antibody (Pharmingen, USA), and peroxidase-conjugated streptavidin were added serially. To develop the second stain, diaminobenzidine substrate (DAB, Pharmingen, USA) was added to the samples. Following that, the cells nuclei were stained using nuclear fast red (Sigma, USA). After each step of immunostaining, the samples were washed with Tris-buffered saline (TBS) three times. Then, the samples were dehydrated by gradient concentrations of ethanol, cleared with Histoclear (Vector Laboratories), and mounted using Vecta Mount (Vector Laboratories).

The NK cells (CD161^+^ and CD3^−^), T cells (CD161^−^ and CD3^+^) and NKT cells (CD161^+^ and CD3^+^), were observed as blue, brown, and mixture of blue and brown colors under light microscope, respectively. The nucleus of cells negative for both markers appeared by counter staining (nuclear fast red). 

For cell counting at least 5 sections in each sample were selected randomly and several microscopic fields in each section were analyzed and the total cells (nuclear fast red stained) and NK cells (blue stained) were counted separately using Image J software. The average of NK cells percent was calculated in each tissue region (stroma and myometrium) in each group of study.

### 2.7. Statistical Analysis

The frequency of NK cells within different parts of the tissue in each group was compared using paired *t*-test. Independent Student's *t*-test was used to compare the proportion of cells in the same region of the tissue between the hyperstimulated and control groups. The sufficient number of our samples was approved by statistically normality test. *P* values of less than 0.05 were considered statistically significant.

## 3. Results

### 3.1. The Sera Estradiol-17*β* and Progesterone Concentrations

The estradiol-17*β* and progesterone concentrations in serum of pregnant mice at seventh day of pregnancy were measured using sandwich ELISA method. The levels of estradiol-17*β* in sera of control and hyperstimulated groups were 87.27 ± 3.86 and 160 ± 3.47 pg/mL and the levels of progesterone in the same groups were 47.55 ± 10 and 164.58 ± 32 ng/mL, respectively. Both hormones levels were significantly increased (*P* < 0.05) in ovarian stimulated group compared to the control mice.

### 3.2. Morphology of the Tissues

**Figure 1:**
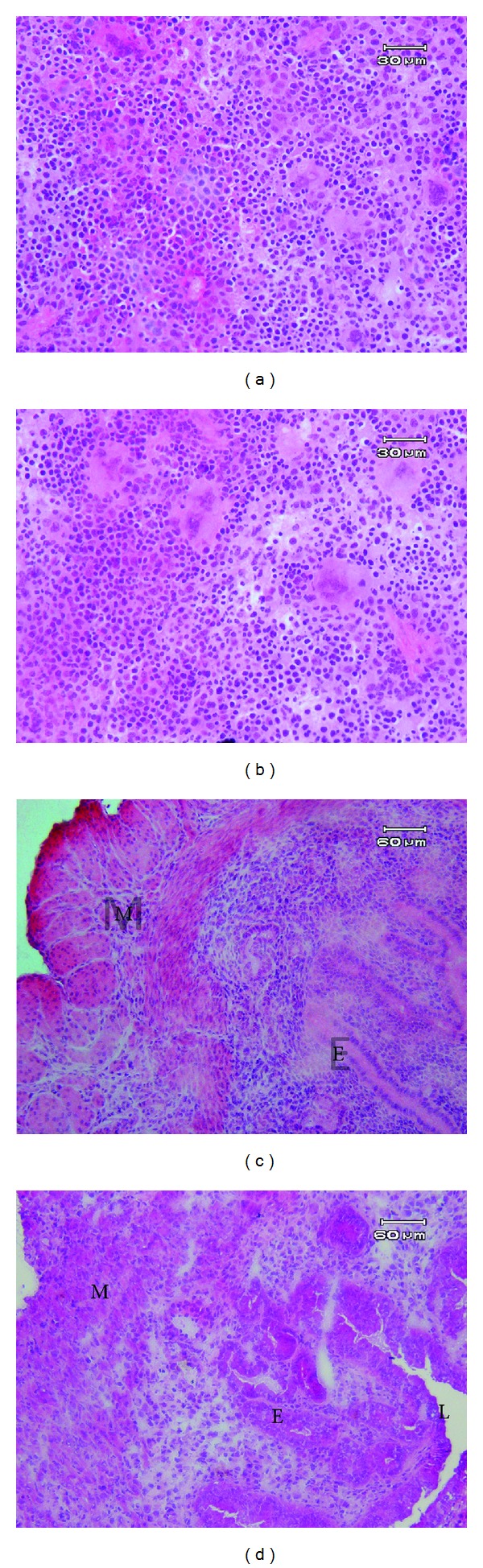
The light microscopic morphology of spleen and uterus tissueS in control (a, c) and hyperstimulated groupS (b, d), respectively, on fifth day of mouse pregnancy. M: muscular layer, S: stroma, E: epithelium, and L: lumen.

The morphology of the splenic and uterine horn tissues that were stained with hematoxylin and eosin did not show remarkable changes between ovarian stimulated and nonstimulated groups (Figure 1). 

### 3.3. Distribution of NK Cells within the Splenic Tissue

**Figure 2:**
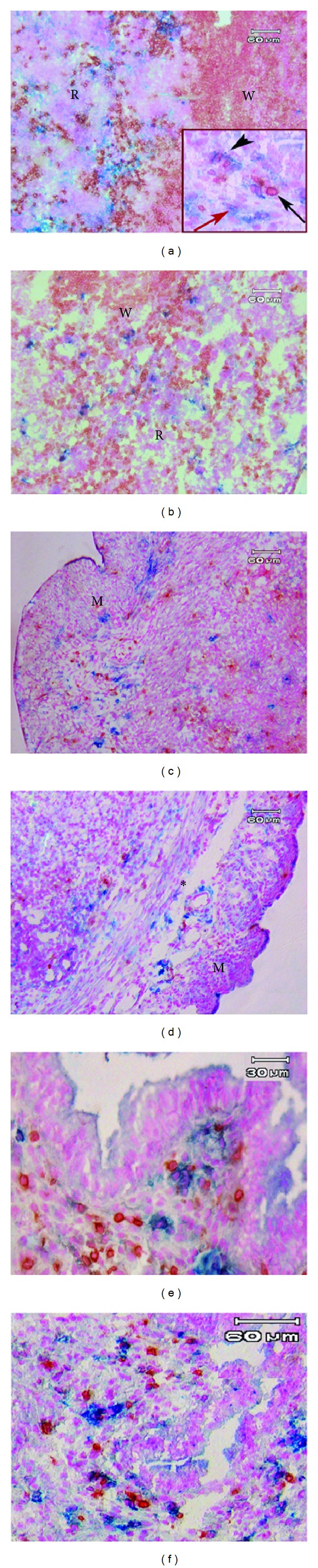
Photograph of double immunohistochemistry staining of splenic and uterine tissue using antiCD3 and antiCD161 antibodies. The spleen of the control (a) and hyperstimulated pregnant mice: R; red pulp, and W; with pulp. The cytoplasm of the immune cells was observed as blue (white arrow) for NK cells (CD161^+^ and CD3^−^), brown (black arrow) for T cells (CD3^+^), and a mixture of blue and brown color (black arrow head) for NKT cells (CD161^+^ and CD3^+^). The uterine horn of control and hyperstimulated groups, respectively, (c and d) at low magnification and in the previous groups (e and f) at high magnification. The lymphocytes and NK cells were prominent around the blood vessel within the muscular layer (∗). M; muscular layer.

**Figure 3:**
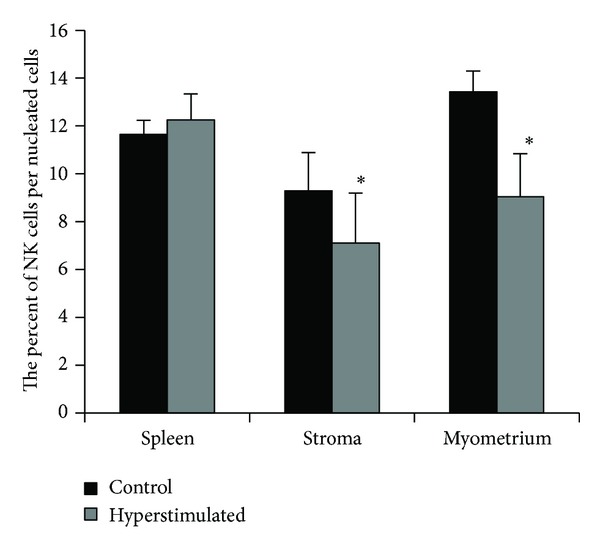
The comparison of the NK cells frequency within the spleen tissue, stroma, and myometrium of the uterine horns in control and hyperstimulated groups. *: showed significant differences with control group (*P* ≤ 0.05).

The results of splenic tissue immunohistochemical double staining with anti-CD3 and anti-CD161 antibodies indicated that the cells, which express CD161 while not expressing CD3 marker (NK cells), are mainly located in red pulp of spleen (Figures 2(a) and 2(b)). Moreover, the mean percentages of NK cells in the splenic tissue were 11.65 ± 0.58% and 12.25 ± 1.09% in the control and hyperstimulated groups, respectively. The difference between the two groups was not statistically significant in this respect (Figure 3).

### 3.4. Distribution of NK Cells in Uterine Horn

The immunohistochemical staining of uterine horns in control and hyperstimulated mice was shown in Figures 2(c)–2(f). The NK cells in the uterine horn tissue were particularly present in the stroma, around blood vessels, and in the myometrium. 

NK cells composed 9.29 ± 1.6% and 7.1 ± 2.1% of nucleated cells of stroma in the control and hyperstimulated groups, respectively (Figure 3). Moreover, the mean percentages of these cells in the myometrium were 13.44 ± 0.86% and 9.04 ± 1.8% in the same groups, respectively (Figure 3). The relative ratio of NK cells was significantly lower not only at the stromal part but also at the myometrium in hyperstimulated than the control group (*P* < 0.05).

The population of NK cells in stroma of control uterine horn was significantly lower than those in the myometrium in this group (*P* < 0.05), but there was no significant difference in this regard in hyperstimulated group.

## 4. Discussion

Uterine natural killer cells are the most frequent population of lymphocytes which are present at the fetomaternal interface during the first half of normal pregnancy in rodent, human, and nonhuman primates [12, 39]. Indeed, there they play important roles to support the differentiation of decidual cell and maintenance of the pregnancy [40]. In this study, using the markers of CD161 and CD3, we studied the localization and relative frequencies of mouse NK cells (CD161^+^ and CD3^−^) in uterine horn and spleen of the nonstimulated and hyperstimulated groups of pregnant mice.

Evaluation of the endometrial NK cells population between the two groups of study demonstrated that these cells are significantly decreased in the stroma and myometrium in the hyperstimulated group. There was poor information about the effects of superovulation on the mouse uterine NK cells population.

It was shown previously by our group [31] and other investigators [30] that supraphysiological levels of ovarian hormones after ovarian induction decrease the embryo quality and implantation rates. Moreover it is suggested that increased levels of ovarian hormones after superovulation may affect the distribution and frequency of uterine NK cells directly or indirectly. In the current study, estrogen and progesterone levels were analyzed in the two groups, and it was observed that the levels of these hormones were significantly higher in the ovarian stimulated group.

It seems that estrogen is one of the main factors in suppressing the proliferation of NK cells as Hao et al. [41] reported that elevation of estrogen level could lead to a decrease in the number of NK cells at the implantation site. 

Furthermore, the level of progesterone in the hyperstimulated group was high, which may have various effects on the NK cells population. The presence of progesterone receptor on the uterine NK cells surface was previously confirmed [21–24]. Adhesion of progesterone to its receptor on the surface of NK cells can activate the caspase signaling pathway in these cells which may lead to induction of apoptosis and decrease of their frequency [24]. The estrogen or progesterone may also indirectly affect the proliferation, migration, and homing of the uterine NK cells progenitors. However, to confirm these suggestions additional studies are needed.

In fact there is some controversy about the exact effects of ovarian hormones on uterine NK cells as DeLoia et al. [19] have shown that the total number of lymphocytes in human endometrium as well as in the peripheral blood, particularly the NK cells, increases under the influence of supraphysiological level of estrogen.

The regional secretion of progesterone by placenta may affect the synthesis and secretion of different local factors which prevents NK cell proliferation and activation [42, 43]. According to the high level of progesterone in hyperstimulated group it seems that this effect is more prominent in this group. Therefore, along with the hormonal changes in mother's circulation, some biochemical changes occur locally at the site of implantation, which influence the implantation of embryo. 

The subtypes of NK cells were not determined in our study so more information is needed to explain the exact mechanism and reason of NK cells reduction in hyperstimulated group compared to the control.

The number of NK cells within the splenic tissue as a representative of their systemic variation was not statistically different in the hyperstimulated group compared to nonstimulated mice. In other words, elevated levels of estrogen and progesterone in blood do not have a considerable effect on the NK cells population of spleen. 

## 5. Conclusion

It seems that ovulation induction procedures could reduce the proportion of NK cells within the uterus, and considering the important role of these cells in establishment of a successful pregnancy, it may affect the development of embryo and implantation.
